# *Paecilomyces variotii*: A Fungus Capable of Removing Ammonia Nitrogen and Inhibiting Ammonia Emission from Manure

**DOI:** 10.1371/journal.pone.0158089

**Published:** 2016-06-27

**Authors:** Zhiyun Liu, Guohua Liu, Huiyi Cai, Pengjun Shi, Wenhuan Chang, Shu Zhang, Aijuan Zheng, Qing Xie, Jianshuang Ma

**Affiliations:** 1 The key laboratory of Feed Biotechnology of Ministry of Agriculture, Feed Research Institute, Chinese Academy of Agricultural Sciences, Beijing, China; 2 College of Animal Science and Technology, Northeast Agricultural University, Harbin, China; MJP Rohilkhand University, INDIA

## Abstract

Ammonia (NH_3_) emissions from animal manure are a significant environmental and public concern. Despite the numerous studies regarding NH_3_ emissions from manure, few of them have considered microbial nitrification approaches, especially fungal nitrification. In this study, a filamentous fungus was isolated from chicken manure and was used for nitrification. The species was *Paecilomyces variotii *by morphological characteristics and 18S rDNA gene sequencing. It played the biggest role in the removal of ammonium at pH 4.0–7.0, C/N ratio of 10–40, temperature of 25–37°C, shaking speed of 150 rpm, and with glucose as the available carbon source. Further analysis revealed that all ammonium was removed when the initial ammonium concentration was less than 100 mg/L; 40% ammonium was removed when the initial ammonium concentration was 1100 mg/L. The results showed that the concentration of ammonia from chicken manure with strain *Paecilomyces variotii* was significantly lower than that in the control group. We concluded that *Paecilomyces variotii* has good potential for future applications in in situ ammonium removal as well as ammonia emissions control from poultry manure.

## Introduction

Ammonia (NH_3_) emissions are a public and environmental concern in part because they harm human and animal health, but also because ammonia forms small aerosol particles and causes ecosystem acidity [1]. Farming is the primary source of atmospheric NH_3_ especially in areas with intensive livestock farming [2], and domestic animal manure storage and application areas are the most important sources of NH_3_ [2,3]. Therefore, reducing NH_3_ emissions from manure is an urgent issue to protect the environment and maintain health.

Extensive studies on techniques to control NH_3_ emissions from animal manure have focused on in vivo and in vitro aspects. Nutritional means [4,5], functional additives and probiotics [6,7] are the most common strategies used as in vivo tools to reduce NH_3_ emissions. In vitro, physicochemical processes and biological treatments are available to remove NH_3_. Over the last decades, many studies have described effective microorganisms to remove NH_3_. This processing method is promising because it has low capital and operating costs, high removal efficiency [8,9].

Ammonium (NH_4_^+^-N) is one of the compositions of fecal nitrogen. The concentration of dissolved NH_3_ is in dynamic equilibrium with NH_4_^+^ on the surface of the liquid phase of animal manure [2,3]. When this balance is altered slightly, NH_3_ volatilisation will be significantly affected. The reduction in NH_4_^+^-N concentration from manure can change this balance, reduce the dissolved NH_3_ concentration, and further decrease the NH_3_ emissions. Microbes that oxidize ammonia play a crucial role during the NH_4_^+^-N conversion process. However, few studies have been conducted on reducing NH_3_ volatilisation through microbial nitrification [9], especially fungal nitrification.

Recent studies have highlighted the NH_4_^+^-N removal capacity of some bacteria including *Rhodococcus* sp. [8], *Agrobacterium* sp. [10], *Alcaligenes faecalis* [11], *Pseudomonas stutzeri* [12], *Halomonas campisalis* [13], and *Providencia rettgeri* [14]. Under certain conditions, these heterotrophic microorganisms oxidize ammonia to N_2_O and/or N_2_. It is one of the most economical and attractive methods for environmental remediation [8,9]. Nevertheless, it has been shown that fungi are also capable of NH_4_^+^-N removal at various substrate concentrations and play an increasingly dominant role in nitrogen cycling [15,16]. As early as the 1970s and '80s, some scholars have reported that fungi are heterotrophic nitrifiers with good throughput and efficiency [17,18]. However, only limited studies have been performed on the NH_4_^+^-N removal capacity of fungi despite the very promising results shown in NH_4_^+^-N degradation. Fungi grow slowly in general, but can degrade a greater variety of pollutants and can withstand harsher conditions [19,20,21]. For example, some slurry ingredients such as butyric acid inhibit bacterial activity [22], but fungi can still thrive under these conditions [16]. In our study, the filamentous fungus *Paecilomyces variotii* named HL was isolated and investigated.

The *Paecilomyces variotii* strain has been studied as an efficient tool to degrade aromatic compounds [19,23]. However, no studies have yet reported *Paecilomyces variotii* as a tool to remove NH_4_^+^-N so far. In this study, the fungus *Paecilomyces variotii* was isolated from chicken manure and demonstrated an amazing ability to remove NH_4_^+^-N. The main objective of this work was to evaluate the NH_4_^+^-N removal ability of this strain and analyse the related factors affecting its performance. Studies were also done to evaluate the effect that strain HL had on NH_3_ emissions from chicken manure. This work will serve as a baseline for further studies on the practical applications of *Paecilomyces variotii* HL.

## Materials and Methods

### Isolation of aerobic denitrifying organisms

A basal medium [11] for the enrichment and isolation of aerobic denitrifying organisms contained the following in units of g/L distilled water: glucose 1.25, (NH_4_)_2_S0_4_ 0.25, NaCl 1.00, K_2_HP0_4_·2H_2_0 0.50, KH_2_PO_4_ 0.25, MgS0_4_·7H_2_0 0.25, and trace elements solution (TES) 1.00 mL at pH 7.2–7.4. The concentrations of (NH_4_)_2_S0_4_ and glucose were adjusted according to the experimental design. A TES [24] contained the following in g/L of distilled water: EDTA·2Na 57.10, ZnSO_4_·7H_2_O 3.90, CaCl_2_·2H_2_O 7.00, MnCl_2_·4H_2_O 1.00, FeSO_4_·7H_2_O 5.00, (NH_4_)_6_Mo_7_O_24_•4H_2_O 1.10, CuSO_4_•5H_2_O 1.60, and CoCl_2_•6H_2_O 1.60 at pH 6.0.

The saline and basal media were autoclaved for 20 min at 121°C. A 250 mL Erlenmeyer flask with 80 mL sterile saline and some sanitized glass beads was inoculated with 10 g of chicken manure from a breeding facility of the Chinese Academy of Agricultural Sciences (CAAS, Beijing, China) with permission and witness of the staff–in-charge of the breeding facility and oscillated on an incubator shaker at 180 rotations per minute (rpm) for 30 min. After 20 min of standing, 1 mL of the supernatant was transferred to basic medium and cultured at 30°C for 2 days. One mL of the above cultures was transferred to another fresh basal medium and cultured for 2 days in the same conditions. This procedure was repeated 10 times. The final enrichment culture was streaked on a potato dextrose agar medium plate (PDA, 200 g of peeled potato, 20 g of glucose, and 20 g of agar in 1 L of distilled water) and incubated at 30°C for 3–4 days. The colonies with different morphological characteristics were isolated and purified by serially streaking on the basal medium plate. These strains were activated using PDA liquid medium for 24h at 30°C. Then 5.0 mL bacteria solutions were transferred to sterile centrifuge tube separately and centrifuged 10min at 40 00r/min. After centrifugation the supernatants were discarded and the thalli were washed three times with sterilizing saline respectively. Strain suspensions made with sterilizing saline were inoculated into 50 ml basal medium separately and cultured using shaking culture method at 30°C with shaking speed of 150r/min for 24h. After the culturing, ammonium concentrations of all culture media were measured and compared. The strain with the highest efficiency on ammonia nitrogen-removal was the target strain.

### Preparation of spore suspension and hyphae suspension

The spore suspension and hyphae suspension of the target strain were separately used as a seed solution for the following shaking-culture experiments. Preparation of spore suspension: the isolate was cultured on Czapek Dox agar (CzA, 30 g of sucrose, 3 g of NaNO_3_, 1 g of K_2_HPO_4_, 0.5 g of MgSO_4_·7H_2_O, 0.5 g of KCl, 0.01 g of FeSO_4_·7H_2_O, and 20 g of agar in 1 L of distilled water) at 30°C for conidia. The conidia were then diluted with sterilized saline. For the hyphae suspension, the HL strain was first inoculated in the CzA culture media at 150 r/min at 30°C for 3–4 days. The enriched culture was harvested by filtration with aseptic gauze. The liquid medium was discarded to isolate the mycelium. The mycelium was washed twice with sterile water. It was ground into hyphae suspensions with sterile water at 400 NTU (Nephelometric Turbidity Unit) [25] by sterile ceramic mortars.

### Identification of the selected strain HL

The selected strain HL was identified by morphological and physiological characteristics, as well as 18S rDNA gene sequence analysis. Strain HL was inoculated into Czapek Dox agar medium and cultured at 30°C for 3–4 days to observe the morphological characteristics. The 18S rDNA were amplified by PCR using the fungal common primers (NS1 5’- GTAGTCATATGCTTGTCTC- 3’ and NS8 5’-TCCGCAGGTTCACCTACGGA- 3’) [26]. PCR products were purified and then sequenced by Tsingke biotechnology Beijing Co., (Beijing, China). The sequence was compared with those of other microorganisms in the GenBank database using online Basic Local Alignment Search Tool program [13]. The phylogenetic tree was constructed using MEGA 6.0 by the neighbor joining method.

### Assessment of Ammonia Nitrogen Removal

Ammonium sulphate was used to elucidate the NH_4_^+^-N removal capacity of the isolate. The NH_4_^+^-N concentration was adjusted to 50 mg/L and carbon to nitrogen (C/N) mass ratio was adjusted to 10 with glucose as the carbon source. A spore suspension (10^6^ spores/mL) was inoculated into the 80 mL culture medium in a 250 mL Erlenmeyer flask at a rate of 1%. The medium was incubated at 30°C with 150 r/min shaking speed for 24 h. We sampled every 3 hours during incubation and measured the changes in NH_4_^+^-N, dry cell weight (DCW), NO_2_-N and NO_3_-N concentration. The operation was carried on under the sterile condition throughout.

### Ammonia nitrogen removal at various nitrogen concentrations

We investigated the NH_4_^+^-N removal capacity at various nitrogen concentrations (from 10 to 1100 mg/L). In addition, the initial NH_4_^+^-N concentrations were adjusted to 300 and 1100 mg/L, respectively. These were sampled periodically to determine the changes in concentration of NH_4_^+^-N and DCW after inoculation with strain HL. The glucose content was varied accordingly to keep the C/N ratio at 10. Other culture conditions were the same as above.

### Single-factor experiments to study the factors influencing the NH_4_^+^-N removal capacity of strain HL

Five single-factor experiments were conducted to study the characteristics of strain HL under different culture conditions, including pH, temperature, shaking speed, carbon sources and C/N, respectively. All operation was finished under the sterile condition.

For pH factor, the initial pH was adjusted to 4, 5, 6, 7, 8, and 9 respectively using 1 mol/L HCl or 1 mol/L NaOH solution with constant shaking speed of 150r/min and temperature of 30°C. For temperature factor, the culture temperature was set to 20, 25, 30, 37, and 42°C separately with constant shaking speed of 150r/min and pH of 7. For shaking speed factor, shaking speeds were adjusted to 40, 70, 120, 150, 180, and 210 r/min respectively with constant temperature of 30°C and pH of 7. In the three experiments above, the initial nitrogen concentration was 50 mg/L and C/N was 10 with glucose as carbon source.

In carbon source experiments, sucrose, sodium acetate, starch, sodium citrate, mannitol, and maltose were employed as the sole carbon source instead of glucose in the basal medium, respectively. The amount of each carbon source was determined by fixing the C/N ratio (w/w) at 10 versus a constant ammonia nitrogen concentration of 50 mg/L. In the C/N ratio experiments, the content of the carbon source (glucose) was changed to adjust the C/N ratio to 5, 10, 20, and 40; the nitrogen concentration was fixed at 50 mg/L. The same temperature (30°C), pH (7) and shaking speeds (150 r/min) were maintained in the two experiments above.

All of the above experiments were conducted in triplicate with spore (10^6^ pores/ml medium) inoculum sizes of 1% (v/v). The non-seeded medium and seeded medium without nitrogen were used as controls. Unless otherwise stated, all of the heterotrophic nitrification experiments were conducted for 24 h.

### Ammonia emissions evaluation of chicken manure with strain HL

Chicken manure (100 g) was put into a 1 L beaker, and hyphae suspensions were inoculated at 5% (v/m) with intensive mixing. All beakers contained a 50 mL beaker with 20 mL of 0.01 mol/L sulfuric acid as the absorption liquid. The beaker was then sealed with plastic wrap and cultured at room temperature (about 25°C). Beakers filled with a mixture of chicken manure and equal amounts of distilled water were used as a control group and cultured under the same conditions. The concentration of NH_3_ in the absorption liquid was measured at 24 h, 48 h, 72 h, and 96 h separately. All treatments were carried out in triplicate.

### Analytical and statistical methods

The NH_4_^+^-N was measured by salicylic acid-hypochlorite spectrophotometry; NO_2_^-^-N was determined with an N-(1-naphthyl)-1, 2-diaminoe-thane dihydrochloride spectrophotometric method; NO_3_^-^-N was measured with an ultraviolet spectrophotometric method [27]. The DCW was determined by weighing the mycelium pellet after drying to a constant weight at 105°C [11].

Results were presented as mean values with the Standard Error. The data were subjected to a one-way variance analysis (ANOVA) and independent T-test. Microsoft Excel 2010 and SPSS 19.0 were used to analyse the differences in each parameter among the treatment groups.

## Results and Discussion

### Identification of strain HL

**Fig 1:**
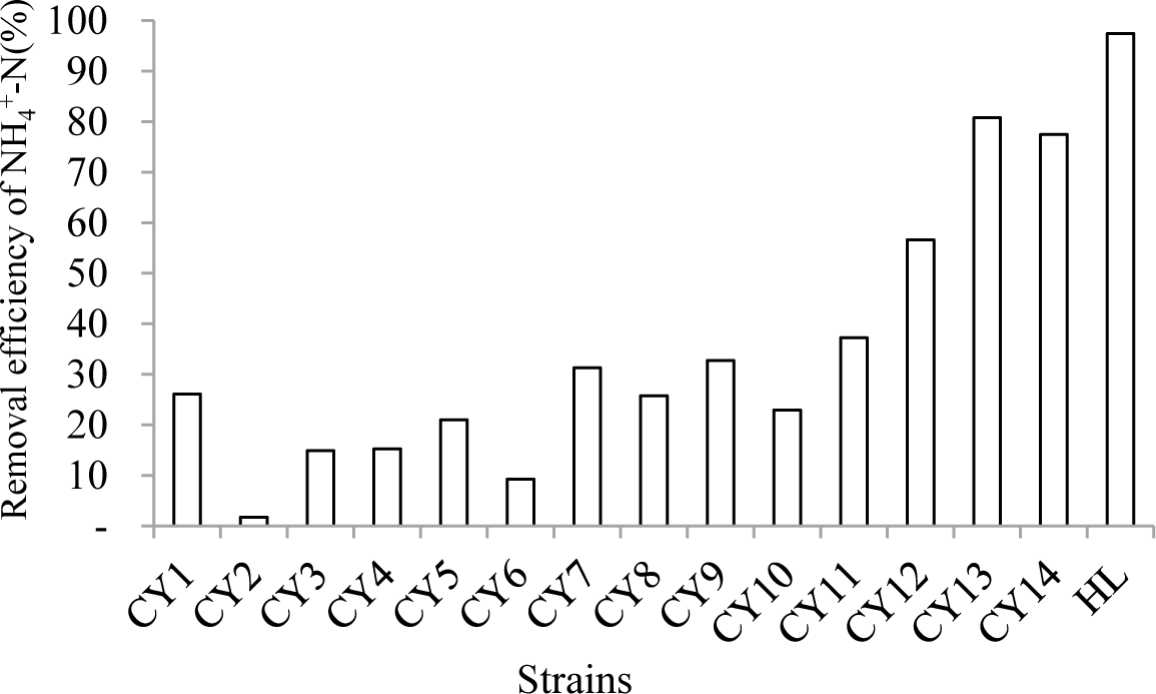
Removal efficiency of NH4+-N of different strains.

There were 15 colonies growing on the enrichment medium. All of them were tested for the ability to remove NH_4_^+^-N (Fig 1). Of these, strain HL removed the most in the least amount of time. Colonial morphology was observed by plating on CzA, and conidia were observed under an optical microscope. Strain HL was recognized as a mycelial fungus. The colonies grew rapidly and formed a dense carpet. The margin was hyaline and fimbriate, while the central part was straw yellow and became dry and powdery in age. Soon after, the central yellow part extended and the colour depth darkened from Naples yellow, straw yellow, olivaceous brown, to brown step-by-step. Similar changes occurred in the reverse of the colony. Conidia were ellipsoidal and smooth, and adhered to long chains. In shake-flasks, strain HL grew mainly in the form of pellets.

**Fig 2:**
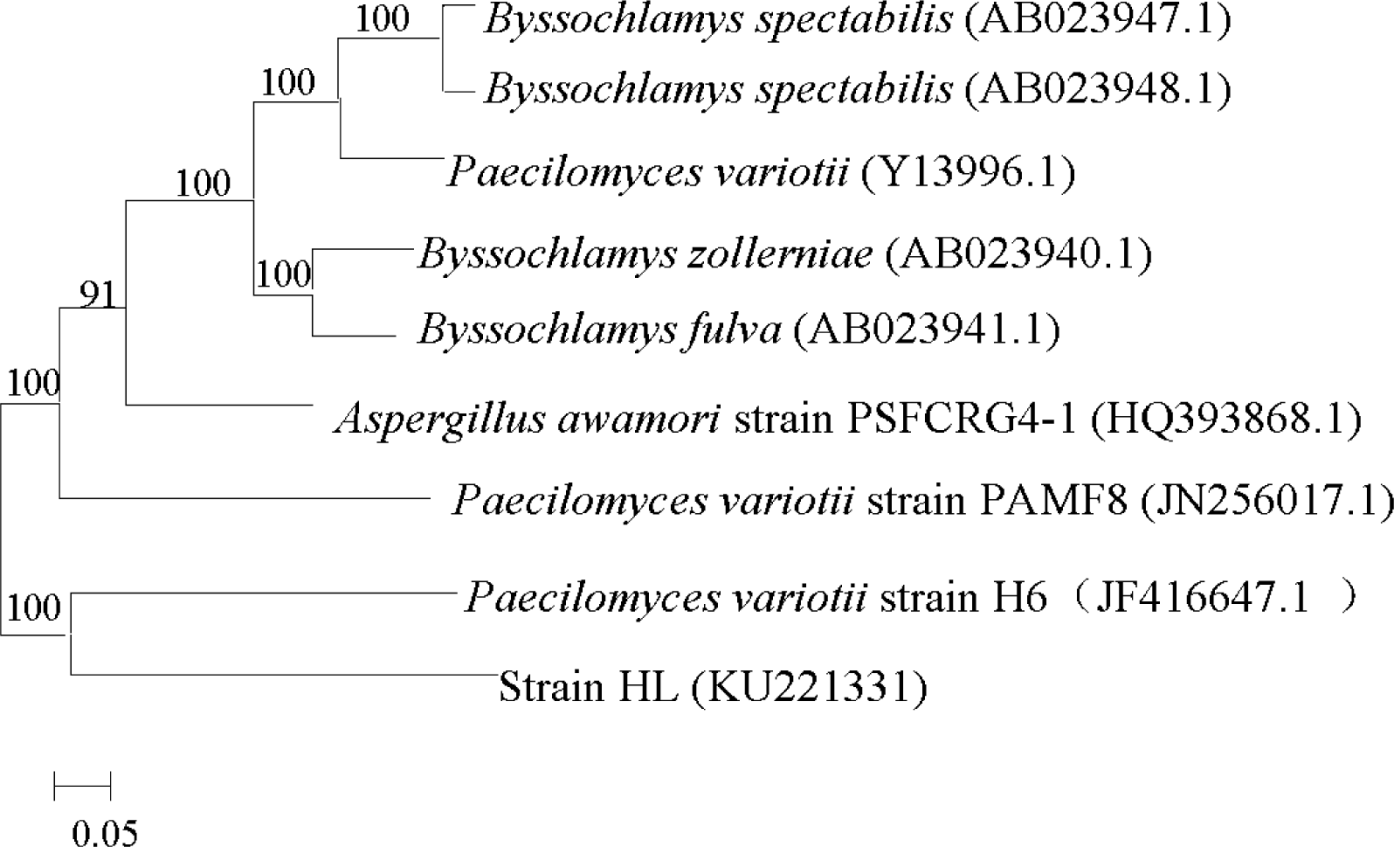
Phylogenetic tree based on the 18S rRNA gene sequences of strain HL. The tree was constructed using neighbor joining method with Bootstrap values of 1000 replications.

An 18S rDNA fragment of 2042 bp was sequenced and 99% sequence similarity with 18S rDNA of *Paecilomyces variotii* was seen versus homologous genes in GenBank using BLAST. Combining the results above, strain HL was identified as *Paecilomyces variotii* (Genbank accession number No. KU221331) and named as *Paecilomyces variotii* HL. The strain had been preserved in the China General Microbiological Culture Collection Center and the preservation number is CGMCC No. 9534. The phylogenetic tree was constructed using MEGA 6.0 by the neighbor joining method (Fig 2).

### The ability of strain HL to remove ammonia nitrogen

**Fig 3:**
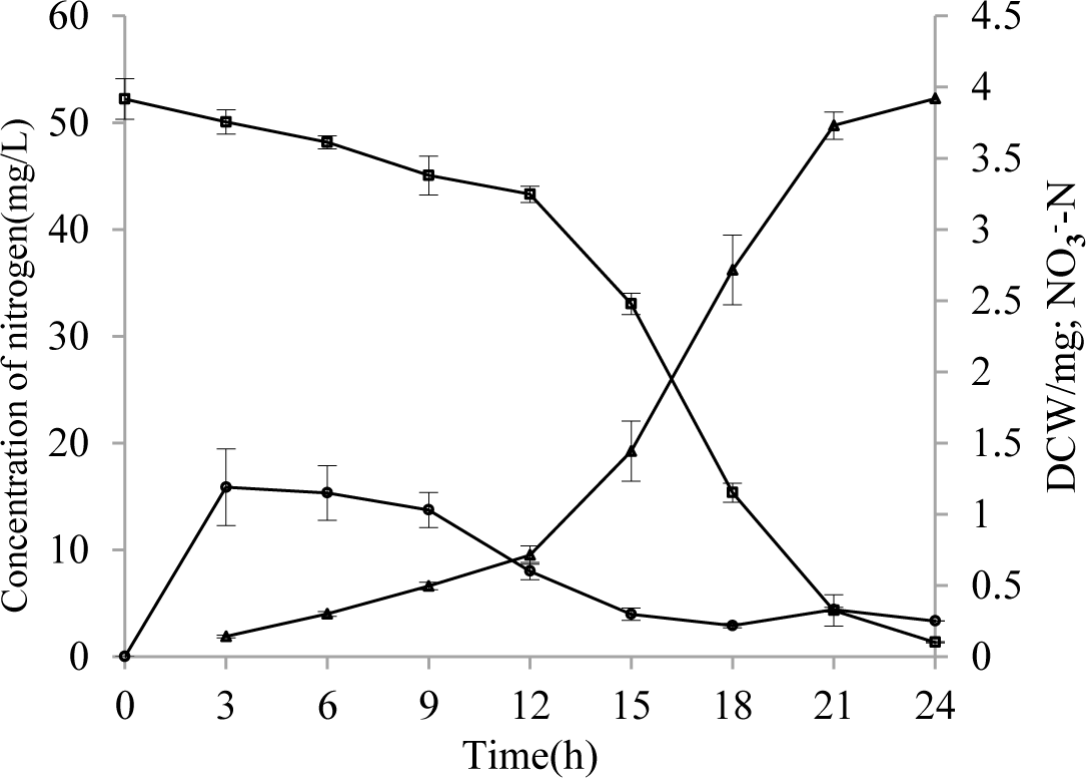
The heterotrophic nitrification of strain HL. Symbols: open squares, NH_4_^+^-N; open circle, NO_3_^-^-N; open triangles, DCW. Error bars: mean ± S.E. of three replicates.

The purpose of the quantitative experiments was to test the capability of strain HL to remove NH_4_^+^-N. When the initial NH_4_^+^-N concentration was 50 mg/L, the residual concentrations were 0 mg/L—a 100% removal rate after 24 h of incubation with little nitrite (NO_2_-N) and only slight nitrate (NO_3_-N) detected in the medium (Fig 3).

The removal rate with strain HL was slow during the first 12 h. A fast removal period was from 12 to 21 h. This corresponded to the logarithmic phase of strain HL. Little NO_2_-N and only slight NO_3_-N was detected. This suggests that strain HL also could perform aerobic denitrification. However, we were unable to identify the exact mechanism as of this publication.

### Ammonia nitrogen removal at various nitrogen concentrations

In a real environment, the substrate (NH_4_^+^-N) concentration is generally uncertain and unstable. Thus research into the tolerance of strain HL for various substrate concentrations is necessary. Ammonia sulphate was used as the nitrogen source to investigate the effect of nitrogen concentrations on the NH_4_^+^-N depletion rate in the basic medium with a C/N value of 10 at 30°C (Table 1). The results showed that when the initial concentration of ammonia nitrogen was less than 110 mg/L, the removal percentage reached 100% in 24 h. The removal percentage decreased from 67 to 40% with the initial concentration of ammonia nitrogen from 177 to 1098 mg/L, respectively. In other words, strain HL could completely metabolize NH_4_^+^-N at lower (<110 mg/L) initial NH_4_^+^-N concentrations, and the degradation was incomplete at higher (177–1098 mg/L) concentrations. The maximum removal rates shown in Table 1 indicated that the removal amount increased with increasing initial NH_4_^+^-N concentrations. This suggests that high NH_4_^+^-N loads would not inhibit the ammonium removal activity of strain HL.

**Table 1:** Ammonium removal capability of HL at various nitrogen concentrations.

Cultivation time (h)	Initial NH_4_^+^-N (mg/L)	Final NH_4_^+^-N (mg/L)	Removal Percentage (%)	Maximum removal Rates (mg-N/L/h)
**12**	11±0.64	0	100	0.93
**24**	54±0.58	0	100	2.67
**24**	110±1.45	0	100	4.59
**48**	177±0.81	58±5.17	67	4.97
**60**	226±2.56	12±1.8	75	5.01
**60**	444±5.66	71±5.95	80	8.89
**60**	631±2.24	130±4.15	68	13.24
**84**	1098±8.27	662±0.99	40	14.75

Yang et al (2011) [28] reported that *Bacillus subtilis* A1 could remove 58% of NH_4_^+^-N within 60 h at an initial concentration of 104 mg/L indicating the incomplete degradation of strain A1. This was similar to our result. However, Joo et al (2005) [11] reported the opposite effect when they studied the ammonium removal ability of *Alcaligenes faecalis* No. 4. They found that the removal percentage of NH_4_^+^-N under low, intermediate, and high ammonium loads all reached 100% in 93 h. These discrepancies might be attributed to the different metabolic pathways and endurance abilities of the high ammonium loads with different microbes. It also indicated that the initial nitrogen concentration was a critical factor in controlling the degradation of ammonium. In this study, we demonstrated that strain HL could tolerate a broad range of ammonium concentrations from 10 mg/L to 1100 mg/L. This could be advantageous in wastewater treatment, especially industrial or agricultural wastewater systems with high ammonium loads [14].

**Fig 4:**
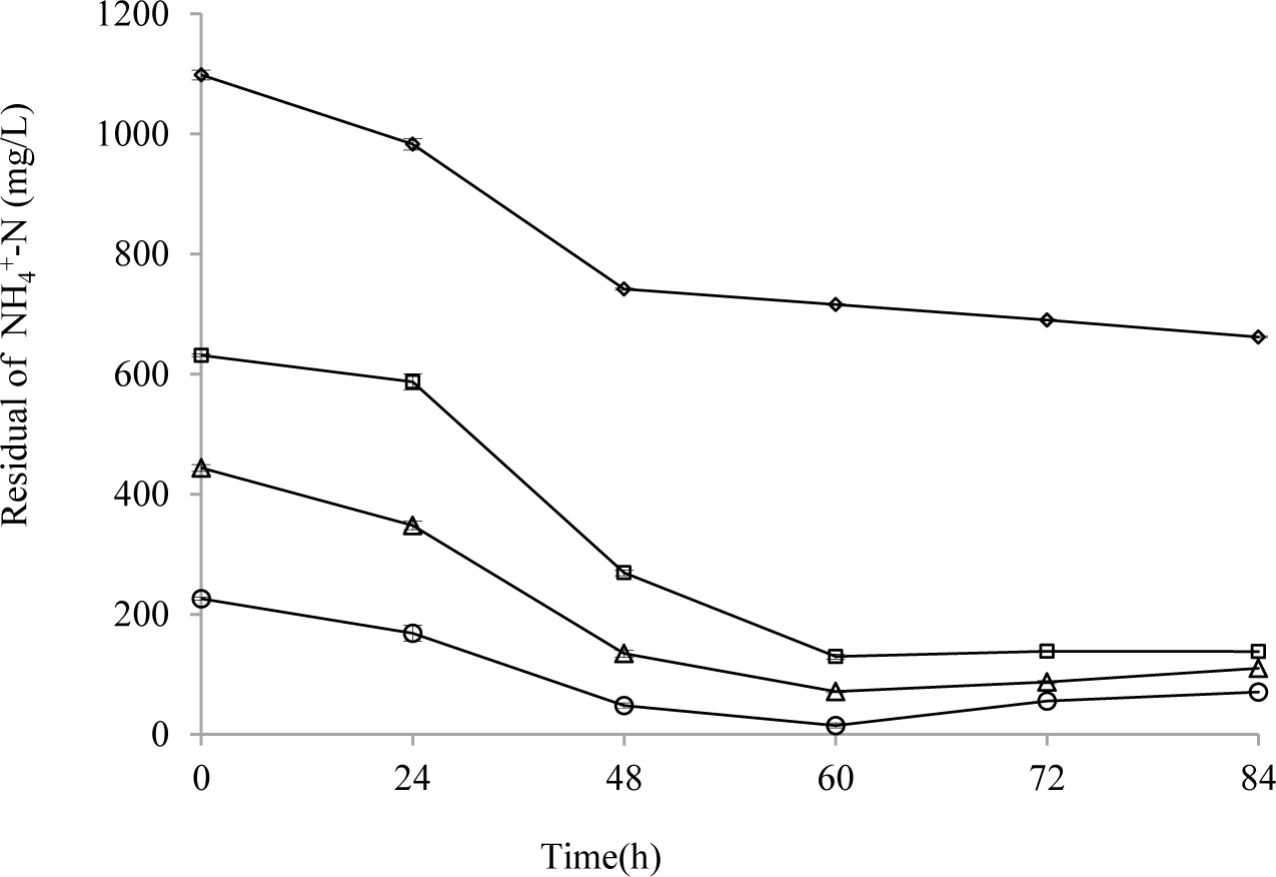
Nitrogen removal capability of strain HL at various concentrations of ammonia nitrogen. Symbols: open rhombus, initial NH_4_^+^-N concentration 1100 mg/L; open squares, 600 mg/L; open triangles, 400 mg/L; open circle, 200 mg/L. Error bars: mean ± S.E. of three replicates.

Fig 4. provides further indications of the changes in NH_4_^+^-N at various initial concentrations during an 84 h growth period. The concentration of NH_4_^+^-N was changed little after 60h. It suggested that strain HL may be limited by some factors, such as some nutrients or some metabolites.

**Fig 5:**
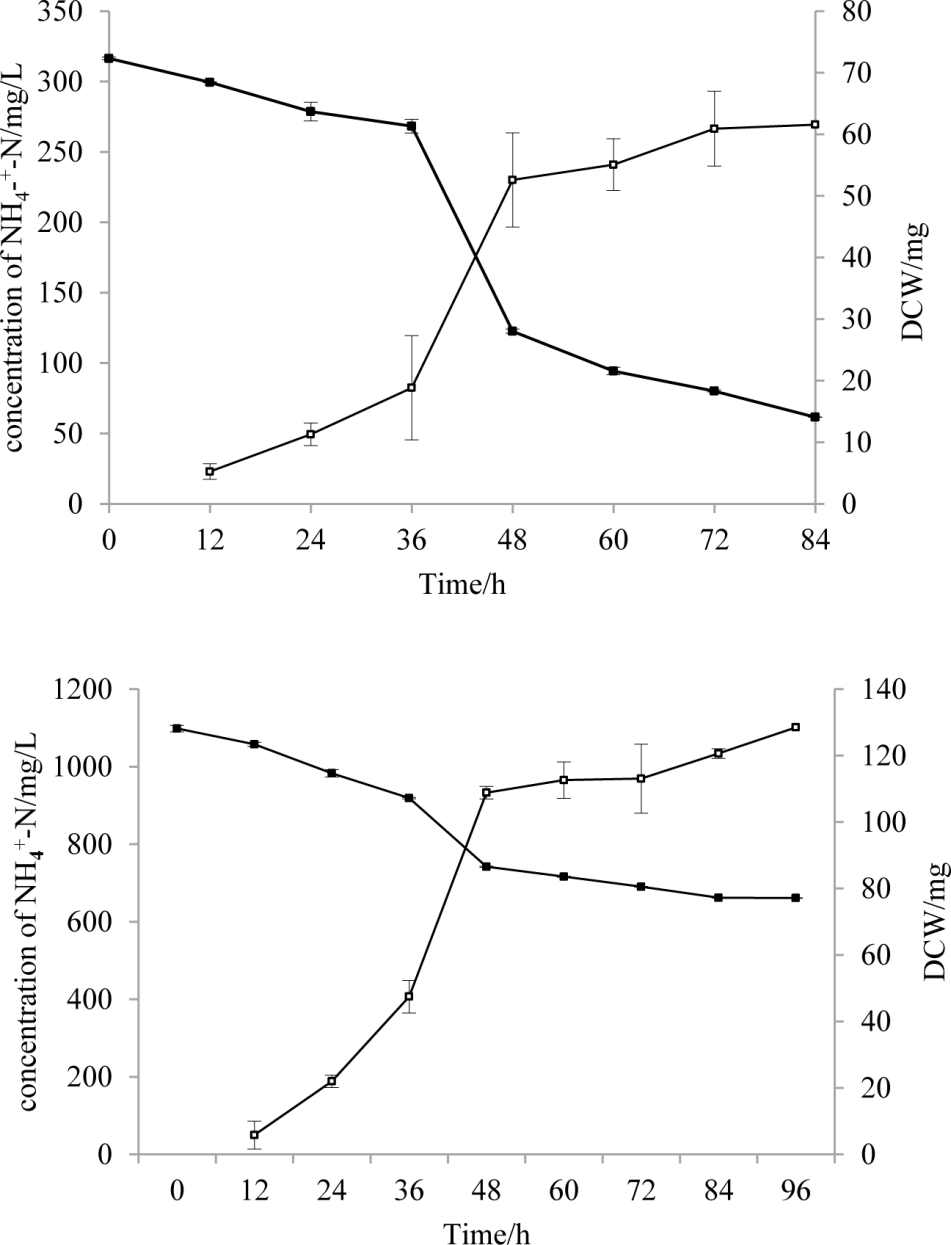
**Changes in DCW and NH**_**4**_^**+**^**-N concentrations by strain HL in a basal medium of 300 mg/L (A) and 1100 mg/L (B) NH**_**4**_^**+**^**-N.** Symbols: open squares, DCW; solid squares, NH_4_^+^-N concentration. Error bars: mean ± S.E. of three replicates.

Additional studies were conducted to understand the correlation of cell growth and NH_4_^+^-N degradation. Changes in NH_4_^+^-N concentration and DCW at different times are shown in Fig 5A and 5B. The changed rate of NH_4_^+^-N concentration and DCW in these two media with different initial concentrations were similar overall. Rapid removal of NH_4_^+^-N occurred after 36 h, and moderate removal was observed after 48 h. This corresponded to cell proliferation and stationary phase, respectively. In addition, cell growth was clearly affected by the availability of nitrogen sources. The changes in DCW corresponded to the NH_4_^+^-N concentrations. Low initial NH_4_^+^-N concentrations led to a lower DCW.

### Results of single-factor experiments

#### pH

**Fig 6:**
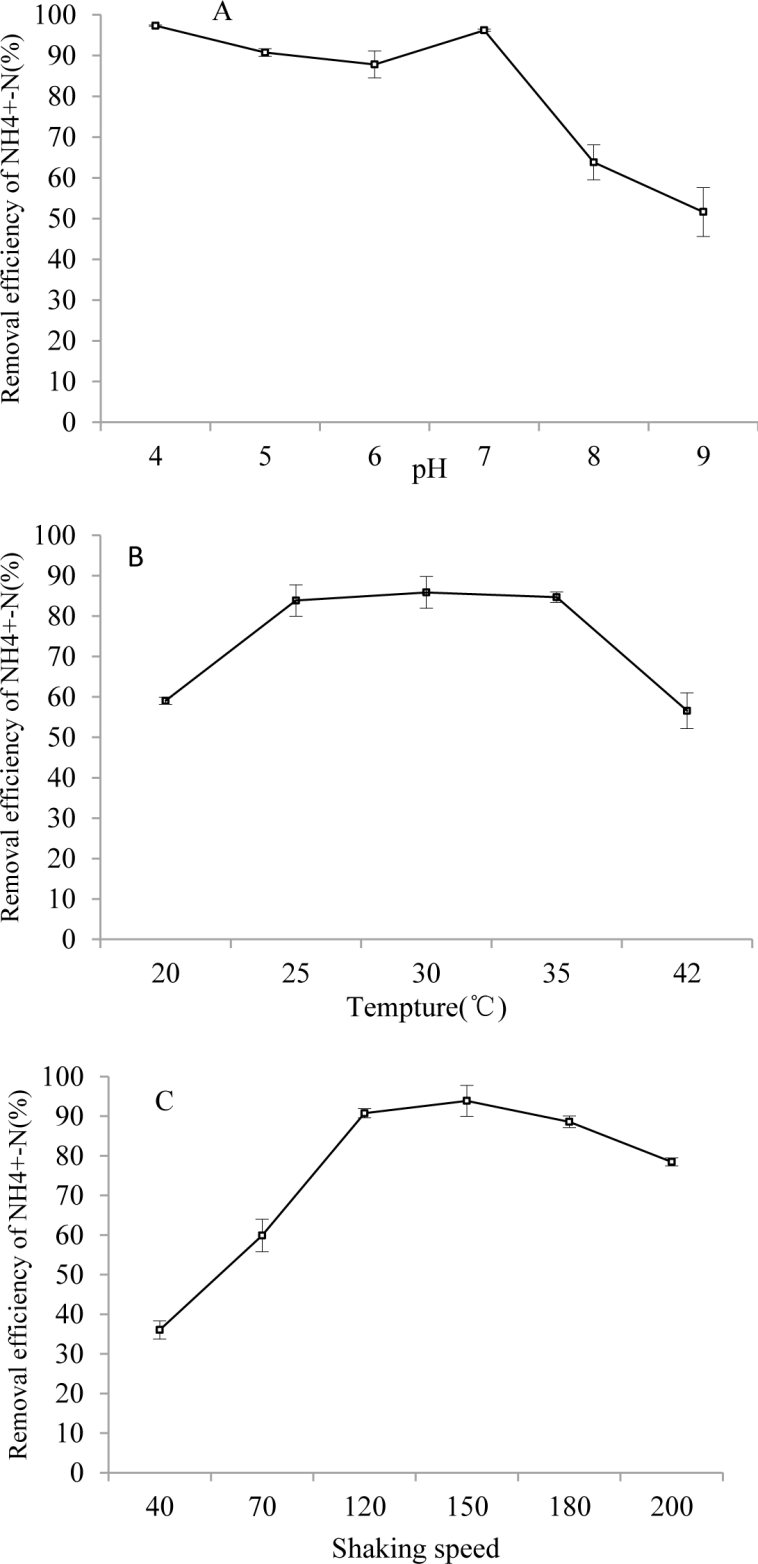
Effects of factors on nitrogen depletion rate by strain HL. Initial pH (A), temperature (B), and shaking speed (C). Symbols: open squares, removal percentage of NH_4_^+^-N. Error bars: mean ± S.E. of three replicates.

The ammonium removal efficiency was affected by changes in pH. When the initial pH of each medium was adjusted to 4, 5, 6, and 7, the NH_4_^+^-N removal of strain HL was 97%, 91%, 88%, and 96%, respectively (Fig 6A). However, when pH reached 8 and 9, the removal efficiencies of NH_4_^+^-N declined markedly indicating that the NH_4_^+^-N removal performance was better under acidic and neutral conditions. This was in contrast to bacterial systems. Many studies have shown that denitrifying bacteria could survival well under slightly alkaline conditions [10,12,29]. For instance, studies developed with *Agrobacterium* sp. LAD9 showed that the optimal pH for NH_4_^+^-N removal was 8.46 [10].

One reason for this discrepancy may be that the optimum pH of most fungi is acidic or neutral—quite different from bacteria. In addition, previous studies have shown that the activities of the ammonia-oxidizing microorganism can be inhibited by high free ammonia (NH_3_-N) [30,31]. Indeed, pH is a key factor that determines the free ammonia concentration in equilibrium with NH_4_^+^-N [3]. When the medium is acidic, the equilibrium moves to the NH_4_^+^-N direction and decreasing free ammonia. This is another explanation why the acidic environment is more conducive to NH_4_^+^-N removal via strain HL. A pH of 7 was deemed appropriate for strain HL to remove NH_4_^+^-N.

#### Temperature

Studies conducted to determine the optimal temperature for ammonium removal revealed that the proper temperature range was 25 to 37°C (Fig 6B). The NH_4_^+^-N removal percentage increased from 59% at 20°C to 86% at 30°C, but decreased to 57% at 42°C. Strain HL had relatively stable removal efficiencies across a wide range of temperatures indicating that this strain had a wider range of tolerance than *Alcaligenes faecalis* No. 4 [11], *Pseudomonas stutzeri* YZN-001 [32], *Providencia rettgeri* YL [14], and *Agrobacterium sp*. LAD9 [10].

Moving away from the ideal temperature in either direction decreases NH_4_^+^-N removal. Lower temperatures inhibit the enzyme activity and reduce the concentration of free ammonia, which is the substrate of ammonia monooxygenase (AMO) [30]. On the other hand, high temperatures also inhibit NH_4_^+^-N removal by increasing the level of free ammonia [30, 31].

#### Shaking speed

Previous studies have indicated that variations in shaking speed remarkable influence the level of dissolved oxygen (DO) in any solution [11,33]. Taylor et al. (2009) showed that DO concentrations increase as a function of shaking speed [14]. In our study, the effect of shaking speed on ammonium removal was investigated rather than DO.

The results showed that the ammonium removal rate increased as the shaking speed increased from 40 (36%) to 150 (94%) r/min (Fig 6C)—the maximum NH_4_^+^-N removal occurred at 150 r/min. The percent of NH_4_^+^-N that was removed decreased from 94% at 150 r/min to 78% at 200 r/min. Decreased removal NH_4_^+^-N may be affected by the dissolved oxygen level in the culture media or possibly linked with shearing of hyphae. The removal rate of NH_4_^+^-N by strain HL could be 36% and 60% with shaking speeds of 40 and 70 r/min.

#### Carbon source

**Fig 7:**
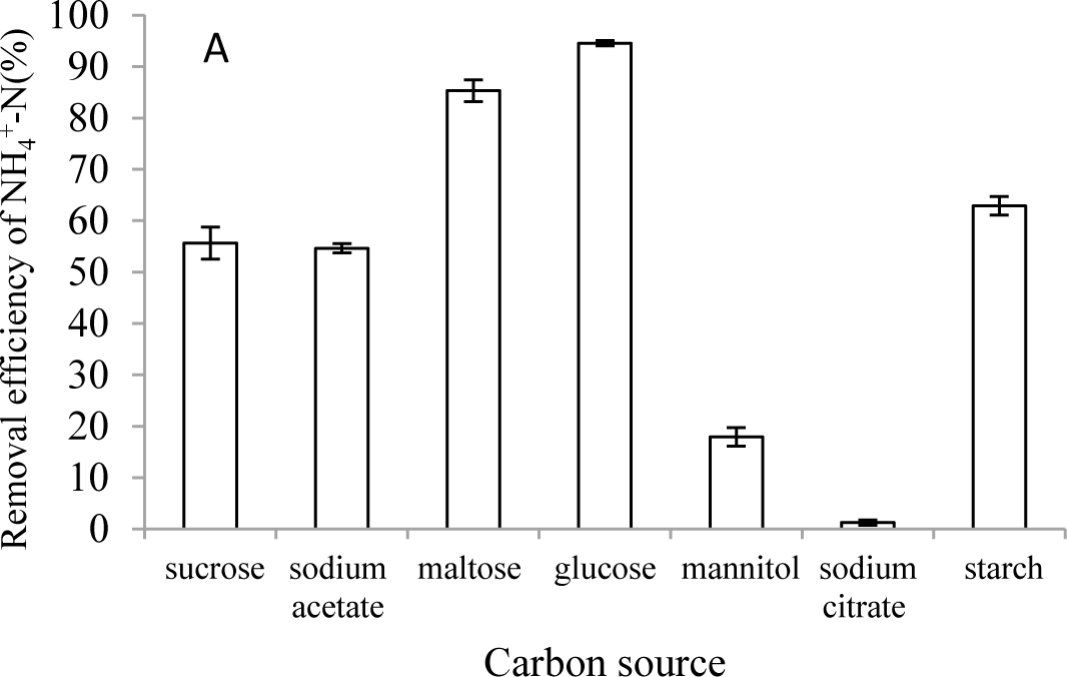
Effects of carbon source on nitrogen depletion rate by strain HL. Carbon source (A). Error bars: mean ± S.E. of three replicates.

The carbon source is an important factor influencing heterotrophic nitrification [10,11,34]. Characteristics of the carbon source had a large effect on the NH_4_^+^-N removal process. A study of 7 carbon sources indicated that glucose was the best for strain HL (Fig 7A). Strain HL had an NH_4_^+^-N removal percentage of 95% with glucose followed by maltose at 85%. Strain HL showed relatively poor nitrification ability with mannital (18%) and sodium citrate (1%) as the carbon sources. This implied that glucose and maltose could support NH_4_^+^-N removal via strain HL.

#### Carbon-nitrogen ratio

The influence of the C/N ratio (5, 10, 20, and 40) on ammonium removal was investigated in basal medium (Table 2). At C/N 5, only 50% and 56% of the NH_4_^+^-N was removed after 12 and 24 h, respectively. This was significantly lower than the other groups (P < 0.01). The NH_4_^+^-N removal percentage was not significantly different at C/N values of 10, 20, and 40 both at 12 h (P > 0.05). The highest removal percentage was 100% at a C/N ratio of 40 within 24 h. A high NH_4_^+^-N removal rate was obtained at C/N 40, which was significant higher than the rate at C/N values of 10, 20, and 40. This indicated that more carbon sources in the medium was needed for the NH_4_^+^-N complete removal. Nishio et al (1998) [35] and Jetten et al (2001) [36] reported that a high C/N was always required because heterotrophic nitrification processes require organics as an energy source for nitrification and as electron donors in denitrification. This implied that strain HL took better advantages of wastewater treatment with a high C/N ratio especially on animal manure mixed with urine and faeces. For example, swine wastewater was reported to usually contain a high C/N ratio (5–20) [37,38]. However, at C/N 5, there was a significant drop in the NH_4_^+^-N removal ability (56%, *p*<0.01) compare with other treatment. Therefore, the addition of external carbon is necessary for efficient wastewater treatment with high NH_4_^+^-N levels and C/N values below 10.

**Table 2:** Effects of C/N ratio on nitrogen depletion rate by strain HL.

Time	Removal efficiency of NH_4_^+^-N (%)				
	C/N 5	C/N 10	C/N 20	C/N 40	*P*-value
12h	50±1.48^b^	68±1.23^a^	64±1.54 ^a^	64±0.42 ^a^	<0.001
24h	56±0.24^d^	93±0.15^c^	94±0.05^b^	100±0.01^a^	<0.001

^a, b, c, d^ means within the same line with different superscripts are significantly different (P<0.05).

### Evaluation of ammonia emissions from chicken manure with strain HL

Though NH_4_^+^-N removal by aerobic heterotrophic nitrifying-denitrifying bacteria in pure culture at the laboratory-scale has been reported multiple times [13,34,39], only limited information is available regarding animal excrement in open mode [38]. In our study, the concentrations of NH_3_ released from chicken manure were monitored periodically throughout the experiment and the data were analysed by independent-samples T test in this part. The NH_3_ removal efficiency of strain HL was evaluated according to the NH_4_^+^-N concentration in the absorption liquid and compared to the control group (Table 3). The emission of ammonia in the experimental group was significantly lower than that of the control group (*P* < 0.05). The removal/ conversion rates in the treatment groups were 54, 39, 79, and 73% at 24 h, 48 h, 72 h, and 96 h, respectively.

**Table 3:** Ammonia emissions from chicken manure treated with strain HL.

Item	NH_4_^+^-N concentration (mg/L)			
	24h	48h	72h	96h
Control group	1.13±0.08^a^	5.43±0.43^a^	11.87±1.36^a^	12.15±3.55
HL group	0.52±0.11^b^	3.30±0.08^b^	2.55±0.91^b^	3.25±0.06
*P* value	0.001	0.001	0	0.054

^a, b^ means within the same column with different superscripts are significantly different (P<0.05).

The results highlight the ammonia control potential and survival capacity of strain HL in chicken manure. However, this is only a 96-hour investigation, and long-term experiments are needed to evaluate the persistence and stability of the treatment efficiency.

## Conclusions

Strain *Paecilomyces variotii* HL can remove ammonium from synthetic medium and reduce the ammonia emissions from chicken manure. The optimum conditions for NH_4_^+^-N removal are pH 4–7, 25–37°C, C/N 10–40, and 150 r/min with glucose as the carbon source. The strain can perform satisfactory ammonium removal across a wide range of initial ammonia concentrations (10 mg/L to 1100 mg/L). The maximum removal rate reached 15 mg-N/L/h. This scheme also reduces ammonia emissions from chicken manure. The maximum removal/ conversion proportion of ammonia was 84%. Future studies will offer a more mechanistic understanding of this process.

## Supporting Information

S1 Table Removal efficiency of NH_4_^+^-N of different strains. (XLSX)

S2 Table Nitrogen removal capability of strain HL. (XLSX)

S3 Table Effects of C/N ratio on nitrogen depletion rate by strain HL. (XLSX)

S4 Table Ammonia emission from chicken manure treated with strain HL. (XLSX)

S5 Table The heterotrophic nitrification of strain HL. (XLSX)

S6 Table Changes in CDW and NH_4_^+^-N concentrations by strain HL in basal medium. (XLSX)

S7 Table Effects of factors on nitrogen depletion rate by strain HL. (XLSX)

S8 Table Effects of carbon source on nitrogen depletion rate by strain HL. (XLSX)
